# ﻿*Primulapingbaensis* (Primulaceae), a new species from Guizhou, China

**DOI:** 10.3897/phytokeys.221.97948

**Published:** 2023-03-10

**Authors:** Na Zhang, Xiao-Qi Jiang, Zhi-Kun Wu

**Affiliations:** 1 Department of Pharmacy, Guizhou University of Traditional Chinese Medicine, Guiyang, 550025, Guizhou, China Guizhou University of Traditional Chinese Medicine Guiyang China

**Keywords:** Guizhou, Karst, new species, ping ba bao chun, *Primula* sect. *Petiolares*

## Abstract

*Primulapingbaensis* Na Zhang, X.Q.Jiang & Z.K.Wu, a new species of Primulaceae from Gaofeng Mountain of Pingba county, Guizhou, China, is described and illustrated. Morphological evidence supports *P.pingbaensis* as a member of P.sect.Petiolares on account of scape elongating, pedicels conspicuously thickening in fruit, and its capsule cracking irregularly round the top and crumbling away. Amongst the members of subsect. Davidii, the new species is characterized by having a uniquely smooth leaf blade due to inconspicuously raised veinlets and homostylous flowers with the style usually extending beyond the anthers. The distribution, phenology and conservation status of the new species are also provided.

## ﻿Introduction

*Primula* L., comprising 521 species mostly found in the temperate zone, is one of the largest genera in Primulaceae (Hu 1994; POWO 2022). More than 300 species of *Primula* were found in China, and distributed mainly in the southwestern part (Yunnan, Sichuan and Tibet), with the modern diversity center of the genus ranging from the Himalaya-Hengduan mountains chain (Hu 1994; Hu and Kelso 1996; Richards 2003).

In *Primula*, floral morph (i.e., heterostyly or homostyly) is one of the important characters for species identification. Heterostyly is perhaps the most distinctive feature of *Primula* (Richards 2003) because most of the species (92%) are heterostylous (Wang et al. 2021). In these species, all the flowers on a plant either have a long style (‘pin morph’) or a short style (‘thrum morph’) differing reciprocally in stigma and anther height. This reciprocal herkogamy is often accompanied by an incompatibility system that makes intermorph crosses more successful than intramorph crosses (Richards 2003). In contrast, the remaining species are almost monomorphic for style condition (i.e., homostylous), having either long styles and long-level stamens (long homostyly) or short styles and short-level stamens (short homostyly). Recently, a novel floral form in *Primula* was reported by one of the authors (Wu et al. 2023), which is characterized by bell-shaped flowers with exceptionally short tubes, stamens set just above the mouth of the tube and the style either as long as, or exserting, the corolla. In addition, some species have both heterostylous and homostylous populations, such as in *P.chungensis* Balf.f. & Kingdon-Ward and *P.oreodoxa* Franch. (Hu 1990).

The Primulasect.Petiolares Pax is well represented in the Himalaya-Hengduan mountains, with only a few members extending into Kashmir, central China, and some other regions (Hu 1990; Hu and Kelso 1996). One of the most important diagnostic characters of the section is that of the capsule being globose, included in the persistent calyx, not opening by valves but crumbling at the membrane apex. More than 60 species of this section are now recognized worldwide (Hu 1994; Hu and Kelso 1996; Hu and Geng 2003; Richards 2003; Li and Hu 2009; Rankin 2010; Hu and Hao 2011; Xu et al. 2014a, 2015, 2016a, 2022a; Ju et al 2018; Yuan et al. 2018).

Guizhou is one of the biodiversity hotspots in China, marked by its karst landscape. According to relevant literatures and Flora of China, there are more than a dozen species of *Primula* distributed in Guizhou, of which some species are endemic to Guizhou, such as *P.esquirolii* Petitm. (Wu et al. 2022), *P.fangingensis* Chen et C.M.Hu, *P.kweichouensis* W.W.Smith, *P.lithophila* F.H.Chen & C.M.Hu and *P.levicalyx* C.M.Hu et Z.R.Xu (Smith et al. 1977; Hu 1990). During the last decade, some newly published *Primula* species from the adjacent areas were also found within Guizhou, such as *P.pelargoniifolia* G.Hao, C.M.Hu & Z.Y. Liu (Xu et al. 2014b), *P.persimilis* G.Hao, C.M.Hu & Y.Xu (Xu et al. 2016b) and *P.centellifolia* G.Hao, C.M.Hu & Y.Xu (Xu et al. 2017). Recently, a newly described species, namely *Primulachishuiensis* C.M.Hu, G.Hao & Y.Xu (Xu et al. 2022b) was reported from Guizhou.

Multiple field surveys were carried out from type locality and adjacent areas from 2018 to 2022 to obtain more reliable information on the field distribution of *P.esquirolii*, species that was last recorded in the 1910s in Pingba county, Guizhou, China. During field surveys in April 2018, we found a *Primula*, which is suspected to be a member of sect. Petiolares with fruits from Gaofeng mountain in Pingba county. At first, we thought it was *P.esquirolii*, but different compared to the type specimen of *P.esquirolii* in its lamina character, where *P.esquirolii* is conspicuously bullate on the upper surface and strongly honeycombed-reticulate beneath. For further clarification on the identity of the newly collected *Primula*, the Gaofeng Mountain and adjacent areas were revisited the following years (2019–2022) to observe and collect the plants in flower and fruit. The collected *Primula* is a slender dwarf perennial herb with smooth, thin papery lamina, bearing homostylous flowers. After comparing the relevant literature for related species, we concluded that this plant was unique and should be described as a new species. Therefore, we describe and illustrate the taxon as new to science here.

## ﻿Materials and methods

The morphological description of the new species was based on living plants from Gaofeng Mountain. For comparison purposes, specimens of closely related species, *P.coerulea* from Yunnan and *P.esquirolii* from Guizhou were also collected from their type locality. In addition, specimens’ images online of the closely related species from herbaria P, E, KUN, PE, K, IBSC, and relevant literature (Smith et al. 1977; Hu 1990; Hu and Kelso 1996) were also consulted. All morphological characters of *P.pingbaensis* and its morphologically similar species in the section Petiolares, including *P.esquirolii* and *P.coerulea*, were measured using a vernier calliper. The conservation assessment of the new species was evaluated using the IUCN categories of threat (see IUCN 2012 and IUCN Standards and Petitions Committee 2022).

## ﻿Taxonomic treatment

### ﻿ Primula pingbaensis

Taxon classificationPlantaeEricalesPrimulaceae

Na Zhang, X.Q.Jiang & Z.K.Wu sp. nov.

01FBABD0-7B9A-53E5-839B-7980D0201AC3

urn:lsid:ipni.org:names:77315510-1

Figs 1A–I , 2

#### Note.

The new species most resembles *Primulaesquirolii* and *P.coerulea*, sharing similar floral morphology and short or almost obsolete scapes at flowering time. However, the new species differs from the homostylous *P.esquirolii* mainly in its lamina smooth on upper surface, deeper flower color, oblong flower lobes, and the style usually extending beyond the anthers. Compared to the heterostylous *P.coerulea*, the new species differs mainly in its homostylous flower, and the whole plant is usually covered with sparse glands. The main morphological distinctions between *P.pingbaensis*, *P.esquirolii* and *P.coerulea* are summarized in Table 1.

**Table 1.** Morphological comparisons of *Primulapingbaensis* with *P.esquirolii* and *P.coerulea*.

Characters	*P.pingbaensis*	*P.esquirolii*	*P.coerulea*
Scape	almost obsolete in flowering time	almost obsolete or to 1 cm in flowering time	apparent scape in flowering time
Leaf blade	2–4 × 2–3 cm, spatulate or elliptic–obovate, thin papery, abaxially sparsely glandular, adaxially smooth	5–13 × 1.5–5 cm, elliptic–obovate to obovate–oblanceolate, subcoriaceous, abaxially densely short glandular pubescent along veins, adaxially bullate	3.5–10 × 2–4.5 cm, elliptic to oblong–oblanceolate, firm papery, abaxially with multicellular hairs along veins, adaxially bullate
Petioles	1/3 as long as leaf blade, sparsely glandular	Short or almost obsolete, sparsely glandular	1–7 cm, with long dense multicellular hairs
Calyx	5–6 mm long	5–7 mm long	8–13 mm long
Calyx lobes	glandular-puberulous, parted nearly to 1/3 of its length	sparsely glandular, parted nearly to middle	pubescent, cut to slightly below middle
Corolla	usually 3 times the length of the calyx, limb 15–20 mm wide, lobes oblong	usually 2–3 times the length of calyx, limb 15–20 mm wide. lobes obovate	nearly one time as the length of the calyx, limb 25–35 mm wide, lobes broadly obovate
Flower color	violet	pale blue or rose or violet	purplish blue
Flower	Homostylous	Homostylous	Heterostylous
Style	14–15 mm long, usually beyond the anthers	style barely reaching base of anthers	Pin flowers: stamens ca. 5 mm above base of corolla tube; style as long as tube. Thrum flowers: stamens 12–13 mm above base of corolla tube; style ca. 6 mm

#### Type.

China. Guizhou: Pingba county, Gaofeng Mountain, Wanhua Temple. 26°22'31"N, 106°24'24"E, 1432 m alt., 14 January 2021 (fl.), *ZKWU 2021010* (holo-type: GZTM!).

#### Description.

A perennial slender, dwarf herbaceous, efarinose, with a short rhizome and numerous fibrous roots. ***Leaves*** forming a rosette, at flowering time 2–4 cm long including the petiole, 2–3 cm broad, spatulate or elliptic-obovate, obtuse or rounded at the apex, gradually tapering into the winged petiole; petiole up to 1/3 as long as leaf blade; lamina thin papery, upper surface smooth, lower surface midrib conspicuous but reticulate veins inconspicuous, sparsely glandular, margin with regular sparsely acute serrate. ***Scape*** at flowering time almost obsolete, to 1 cm in fruit, sparsely glandular, usually 1–3 flowered. ***Bracts*** linear-lanceolate, 3–6 mm long, sparsely glandular; pedicel 5–12 mm, glandular. ***Flower*** homostylous; calyx campanulate, 5–6 mm long, glandular-puberulous, parted nearly to 1/3 of its length, lobes triangular-lanceolate, apex acute; corolla funnel-shaped, violet, tube 13–15 mm long, usually three times the length of the calyx, limb 15–20 mm wide, lobes oblong, 4–6 mm long, apex emarginate; stamens with anthers 1.5 mm long, inserted at apex of corolla tube, style 14–15 mm long, usually beyond the anthers. ***Capsule*** subglobose, 4–6 mm in diameter, nearly equal to calyx.

#### Distribution and ecology.

**Figure 1.**
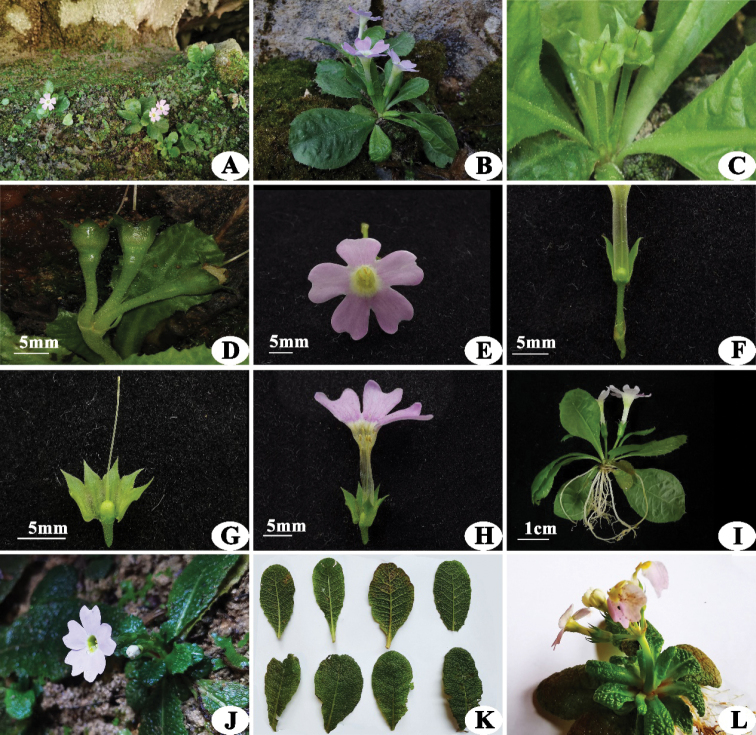
**A–I***Primulapingbaensis* sp. nov.: **A** habitat **B** habit in flowering **C** capsule in early-fruiting **D** capsule in later-fruiting **E** flower, front view **F** bracts and calyx **G** calyx and stigma **H** dissected corolla showing the anthers and stigma **I** specimen in flowering; **J–L***Primulaesquirolii*: **J** habitat **K** leaves, both surfaces **L** habit. Photographed by Z.K Wu.

**Map 1.**
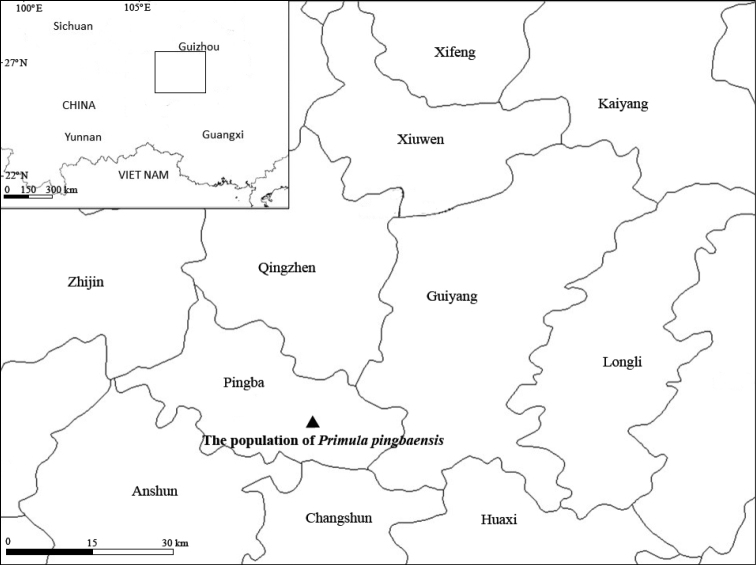
Location of the population of *Primulapingbaensis* in Pingba county.

*Primulapingbaensis* is only known from the type locality on Gaofeng mountain in Pingba county, Guizhou, China. The plant grows on moist walls of karst cliffs. (Fig. 1, Map 1).

#### Phenology.

Flowering occurs from January to March; fruiting from April to June.

#### Etymology.

The epithet of the new species is derived from the name of Pingba county, Guizhou, where the new species was discovered and collected (Map 1).

**Figure 2.**
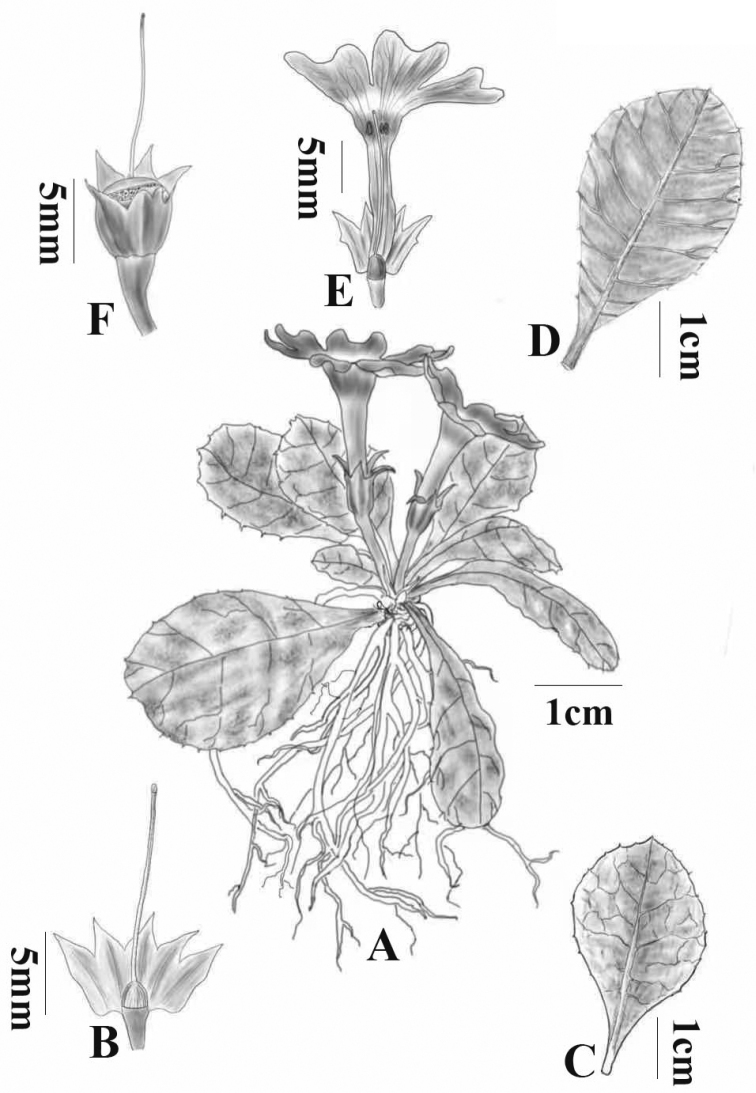
*Primulapingbaensis* sp. nov. **A** habit **B** calyx and stigma **C** upper face of leaves **D** lower face of leaves **E** dissected corolla **F** capsule. Drawn by Ms. Xiang-Li Wu.

#### Vernacular name.

Chinese mandarin: ping ba bao chun (平坝报春).

#### Provisional conservation status.

Critically Endangered (CR B2ab(iii)). Field surveys were conducted several times in the type locality and adjacent districts for this new species, and only one population of *Primulapingbaensis* was discovered, with ca. 40–60 adult individuals, distributed over about 100 m^2^ in the type locality. This site is on the grounds of a temple, and some individuals grow close to the path for visitors and face a strong threat from human activities. Its status should therefore be of concern and addressed in further investigations.

We estimated the extent of occurrence of the species to be less than 100 km^2^. Over the last four years, we have observed a steady decline in the territory area of the habitat due to the temple building maintenance and road construction. Considering the present field information and IUCN categories of threat, this species should be included in the category Critically Endangered (CR B2ab(iii)).

## ﻿Discussion

Most species in the sect. Petiolares of *Primula* have heterostylous flowers. Only *P.hookeri* G.Watt, *P.chamaethauma* W.W.Smith, *P.esquirolii* and our newly discovered species *P.pingbaensis* have homostylous flowers. Species with homostylous morphs are usually accompanied by a compatibility system that makes intramorph (and thus self-) crosses more successful (Richards 2003). The selfing homostylous lineages often have a high genetic load, which makes them sensitive to environmental changes and renders population expansion over short periods difficult (Wu et al. 2022). Our observation on *Primula* species distribution indicated that homostylous species usually have fewer populations or smaller population sizes than closely related heterostylous species. *Primulahookeri* and *P.chamaethauma* are distributed in the alpine areas of Yunnan and Tibet (usually over 4000 m a.s.l.) while *P.esquirolii* and *P.pingbaensis* occur in the karst areas of Guizhou (usually lower than 1500 m a.s.l.). Field surveys on *P.esquirolii* showed that it is a species with extremely small populations and was evaluated as‘Critically Endangered’ (Wu et al. 2022), like our new finding of *P.pingbaensis*.

## Supplementary Material

XML Treatment for Primula pingbaensis

## References

[B1] Hu CM (1990) *Primula*. In: Chen FH Hu CM (Eds) Flora Republicae Popularis Sinicae, Chapter 59.Science Press, Beijing, 1–288.

[B2] Hu CM (1994) On the geographical distribution of the Primulaceae. Journal of Tropical and Subtropical Botany 2(4): 114.

[B3] Hu CM Geng YY (2003) Two New Species of *Primula* (Primulaceae) from China. Novon 13(2): 196–199. 10.2307/3393518

[B4] Hu CM Hao G (2011) New and noteworthy species of *Primula* (Primulaceae) from China. Edinburgh Journal of Botany 68(2): 297–300. 10.1017/S096042861100014X

[B5] Hu CM Kelso S (1996) Primulaceae. In: Wu ZY Raven PH (Eds) Flora of China.Beijing: Science Press & St. Louis: Missouri Botanical Garden Press, 99–185.

[B6] IUCN (2012) IUCN Red List Categories and Criteria. Version 3.1. 2^nd^ edn. IUCN Species Survival Commission, IUCN, Gland, Switzerland and Cambridge, UK.

[B7] IUCN Standards and Petitions Committee (2022) Guidelines for Using the IUCN Red List Categories and Criteria, Version 15.1. Prepared by the Standards and Petitions Committee. [accessed 29. 12. 2022]

[B8] Ju WB Huang Q Sun ZY Huang WJ Li HC Gao XF (2018) *Primulaluteoflora* (Primulaceae), a new species from Sichuan, China. Phytotaxa 367(3): 297–300. 10.11646/phytotaxa.367.3.10

[B9] Li R Hu CM (2009) *Primulalihengiana* (Primulaceae), a new Species from Yunnan, China. Annales Botanici Fennici 46(2): 130–132. 10.5735/085.046.0208

[B10] POWO (2022) *Primula* L. https://powo.science.kew.org/taxon/urn:lsid:ipni.org:names:30005261-2

[B11] Rankin DWH (2010) *Primulanghialoensis*. Curtis’s Botanical Magazine 27(2): 132–139. 10.1111/j.1467-8748.2010.01689.x

[B12] Richards J (2003) *Primula*. 2^nd^ edn. London: Batsford.

[B13] Smith WW Forrest G Fletcher HR (1977) The genus *Primula*. In: Plant Monograph Reprints. Inder A. R. Gantner Vedag Konrnanditgesellsehaft, Cramer J.11: 648–688.

[B14] Wang XJ Barrett SCH Zhong L Wu ZK Li DZ Wang H Zhou W (2021) The genomic selfing syndrome accompanies the evolutionary breakdown of heterostyly. Molecular Biology and Evolution 38(1): 168–180. 10.1093/molbev/msaa199 32761213 PMC7782863

[B15] Wu ZK Wu Y Zhang N (2022) Rediscovery of the critically endangered *Primulaesquirolii*, a karst cave species with an extremely small population endemic to China. Oryx, 1–3. 10.1017/S0030605322001223

[B16] Wu ZK Guo YJ Zhang T Burgess KS Zhou W (2023) *Primulaluquanensis* sp. nov. (Primulaceae), a new species from southwestern China, reveals a novel floral form in the heterostyly-prevailing genus. Plants 12(3): 534. 10.3390/plants12030534 36771618 PMC9918951

[B17] Xu Y Yuan S Hu CM Hao G (2014a) *Primuladejuniana* (Primulaceae), a New Species from Sichuan, China. Annales Botanici Fennici 51(6): 372–374. 10.5735/085.051.0602

[B18] Xu Y Liu ZY Yu XL Hu CM Hao G (2014b) *Primulapelargoniifolia* (Primulaceae), a New Species from Chongqing, China. Annales Botanici Fennici 51(1–2): 25–127. 10.5735/085.051.0117

[B19] Xu Y Hu CM Hao G (2015) A New Species of *Primula* (Primulaceae) from Sichuan, China. Annales Botanici Fennici 23(2): 147–150. 10.5735/085.052.0518

[B20] Xu Y Li CH Hu CM Hao G (2016a) *Primulawawushanica* sp. nov. (Primulaceae) from Sichuan, southwestern China. Nordic Journal of Botany 34(2): 156–158. 10.1111/njb.00894

[B21] Xu Y Yan HF Hu CM Hao G (2016b) *Primulapersimilis* sp. nov. (Primulaceae) from Sichuan, China. Nordic Journal of Botany 34(4): 409–412. 10.1111/njb.01130

[B22] Xu Y Hu CM Hao G (2017) *Primulacentellifolia* (Primulaceae), a new species from south-central China. Phytotaxa 326(4): 259. 10.11646/phytotaxa.326.4.4

[B23] Xu Y He DM Yang LZ Hao G (2022a) *Primulasurculosa* (Primulaceae), a new species from Yunnan, China. PhytoKeys 212: 29–35. 10.3897/phytokeys.212.91133 36761304 PMC9836474

[B24] Xu Y Liu TJ Hu CM Hao G (2022b) *Primulachishuiensis* (Primulaceae), a new species from Guizhou, China. Nordic Journal of Botany 2022(9): e03670. 10.1111/njb.03670

[B25] Yuan S Zhang DX Hao G (2018) *Primulachimingiana* sp. nov. (Primulaceae) from Sichuan, China. Nordic Journal of Botany 36(1–2): 1–4. 10.1111/njb.01390

